# Bridging Tumor Growth Dynamics and Survival in Preclinical Oncology Drug Development – A Translational Tumor Growth Inhibition and Time to Event (TGI-TTE) Modeling Framework

**DOI:** 10.21203/rs.3.rs-9400841/v1

**Published:** 2026-04-23

**Authors:** Kumar Kulldeep Niloy, Jamie Horn, Nazmul Hasan Bhuiyan, Suhas S. Bhosale, Khaled A. Shaaban, Thomas E. Prisinzano, Jon S. Thorson, Jurgen Rohr, Markos Leggas

**Affiliations:** St. Jude Children’s Research Hospital; St. Jude Children’s Research Hospital; University of Kentucky; University of Kentucky; University of Kentucky; University of Kentucky; University of Kentucky; University of Kentucky; St. Jude Children’s Research Hospital

**Keywords:** Tumor growth inhibition modeling, Time-to-Event Modeling, Ewing sarcoma, Mithramycin analogues

## Abstract

**Purpose:**

To develop a translational tumor growth inhibition and time-to-event (TGI-TTE) modeling framework linking drug exposure, tumor dynamics, and survival. Modeling used preclinical efficacy studies with MTMSA-Trp, a preclinical-stage anti-tumor agent for Ewing sarcoma.

**Methods:**

Tumor volume and survival data from Ewing sarcoma mouse xenografts treated with MTMSA-Trp (0.3–2.85 mg/kg) were analyzed. A Simeoni TGI model was used to estimate individual exponential and linear tumor growth rates. A parametric log-logistic TTE model was used to describe mouse survival across treatment groups by incorporating post hoc TGI metrics and regimen-specific average concentrations derived from the PK model as covariates predictive of survival. Models were evaluated using the precision of parameter estimates, goodness-of-fit plots, and bootstraps.

**Results:**

The TGI model accurately described individual tumor growth dynamics, yielding precise parameter estimates (RSEs < 15%). The final TTE model successfully captured the observed Kaplan-Meier curves across treatment groups (RSEs < 20%). Higher regimen-specific average concentration was significantly associated with longer survival (coefficient = 0.63). Conversely, higher exponential and linear tumor growth rates were significantly associated with shorter survival (coefficients = −0.75 and −0.46, respectively).

**Conclusion:**

This framework quantitatively links tumor dynamics, drug exposure, and survival and may support the design, analysis, and simulation-based evaluation of dose regimens in preclinical oncology studies.

## Introduction

To quantitatively evaluate anti-tumor efficacy and characterize the time-course of tumor growth of an anti-cancer agent in mouse xenograft studies, tumor growth inhibition (TGI) modeling has become a common and highly valuable translational approach [1–6]. TGI models describe the tumor growth dynamics of the treated animals by accounting for both natural tumor progression and drug-induced tumor cell kill [7, 8]. However, the translational potential of an anticancer agent requires extending beyond tumor regression to understand its effect on survival [6, 9, 10]. Although TGI modeling is common in preclinical efficacy assessment, the application of integrated TGI-TTE frameworks to preclinical xenograft survival data remains limited.

To bridge this gap, the present work developed a translational TGI-TTE modeling framework to quantitatively link drug exposure, tumor growth dynamics, and survival endpoints as a case study in preclinical oncology drug development. The modeling used data from preclinical studies in Ewing sarcoma (ES) models. ES is a pediatric bone and soft tissue cancer characterized by chromosomal translocation leading to the expression of EWS-ETS fusion proteins, which serve as oncogenic transcription factors driving tumor progression [11, 12]. MTMSA-Trp is an EWS-ETS transcription factor inhibitor currently under preclinical development for the treatment of ES [13–15]. In ES mouse xenograft studies, this compound demonstrated significant tumor regression and improved survival, as measured by predefined tumor volume [14, 15].

In these xenograft studies, survival was defined as time to reach the prespecified tumor-volume threshold, consistent with standard preclinical efficacy practice [9]. Using tumor volume and survival data from ES mouse xenograft studies treated with different MTMSA-Trp dosing regimens, this study employed a two-stage modeling approach. First, a TGI model was developed to estimate individual TGI metrics. Subsequently, a parametric log-logistic TTE model was developed to test these individual post-hoc TGI metrics as well as the drug exposure metric as covariates associated with survival. This framework establishes a quantitative bridge between tumor dynamics and survival, making it more generally applicable to support the design, analysis, and simulation-based exploration of dosing regimens in preclinical oncology drug development.

## Methods

### Animal experiments

Tumor growth data were collected from in vivo tumor growth inhibition experiments conducted in female athymic nude mice bearing TC-32 tumors [14, 15]. Briefly, female athymic nude mice were implanted on the rear left flank by subcutaneous injection of 0.5 × 10^6^ TC-32 cells in 0.1 mL 50:50 RPMI:Matrigel^®^. Mice were monitored for tumor growth using slide calipers and randomly assigned to receive either MTMSA-Trp or vehicle (5%:95% HS-15: PBS, pH 8) via bolus tail-vein injection. The initial tumor volumes ranged from 37 to 251 mm^3^ across three experiments assessing tumor growth inhibition. In experiment 1, mice were dosed at 0.3, 0.6, and 0.9 mg/kg once daily for 5 days (Q1dx5). In experiment 2, mice were dosed at 2.40 mg/kg every 3 days for a total of 6 doses (Q3dx6). In experiment 3, mice were dosed at 2.40 mg/kg for a total of 6 doses (Q3dx6) and at 2.85 mg/kg for a total of 6 doses twice per week (BIWx6). Tumor volumes and animal body weights were measured daily throughout the dosing period, then twice weekly thereafter. Tumor growth data were collected over 100 days across all three experiments. To support estimation of unperturbed tumor growth, vehicle-control data from experiments 1 and 2 were pooled with historical TC-32 control data generated under comparable study conditions.

All animal experiments were performed under an institutionally approved protocol (St. Jude Institutional Animal Care and Use Committee protocol 3164). Female athymic nude mice were purchased from Envigo RMS (Athymic Nude-Foxn1nu; Indianapolis, IN) and Charles River (NU(NCr)-Foxn1nu; Wilmington, MA) for pharmacokinetic and efficacy experiments, respectively. The animals were housed in AAALAC-certified, temperature- and humidity-controlled (64°F to 84°F and 30% to 70%, respectively) facilities with a 12 h light/dark cycle. Mice were provided with standard commercial diets (rodent 5R53 TestDiet, Richmond, IN) and chlorinated, reverse-osmosis-treated water *ad libitum*.

### TGI modeling

The TGI modeling dataset comprised 1,377 tumor volume observations from 104 mice. The TGI model was developed in a stepwise manner. First, a natural tumor growth model was identified using data from the control group. Several structural models describing different growth patterns (e.g., exponential, quadratic, Simeoni, and Koch) were evaluated. The best-fitting natural growth model was then extended to include a drug effect term representing MTMSA-Trp–induced tumor cell kill. The drug effect term included concentrations simulated from a one-compartment PK model with linear elimination, based on previously published PK data [13]. In the PK model-based simulation, the typical population estimate of clearance (CL) was scaled to the dose used in the tumor growth inhibition experiments. TGI model parameters were estimated by simultaneously fitting tumor volume data from both control and treatment groups. Interindividual variability (IIV) on model parameters was assumed to follow a log-normal distribution. Additive, proportional, and combined residual error models were evaluated to characterize residual unexplained variability.

### TTE modeling

A parametric time-to-event (TTE) model was used to estimate the time to tumor volume reaching a predefined survival endpoint of 1,500 mm^3^ for each animal. The TTE dataset included data from experiments 1, 2, and 3 (**Table S1**). Animals that did not reach the endpoint by study termination were censored at their last observation time. Several parametric TTE models, including log-logistic, Weibull, Gompertz, and exponential, were fitted to the survival data from all mice. Upon selection of the base TTE model, covariate modeling was conducted to link TGI and PK parameters to the event time. For this, individual TGI parameters were derived from the TGI model, and exposure was summarized as regimen-specific average concentration ($C_{\text{avg}}$). PK model-derived parameters were used to simulate the concentration-time profile of each regimen, and non-compartmental analysis was used to calculate $C_{\text{avg}}$ from the simulated concentration-time profiles [16].

### Parameter estimation and model evaluation

TGI and TTE modeling and parameter estimation were performed in Monolix version 2024R1 (Lixoft). Model selection was based on the Akaike Information Criterion (AIC), and the model with the lowest AIC was selected as the best fit. Model evaluation was based on the precision of parameter estimates, the visual predictive check (VPC) of 1000 simulations, and the goodness-of-fit (GOF) plots. Model stability was assessed by comparing parameter estimates with those from 1000 bootstrap runs. The data were plotted using GraphPad Prism 10.4.0.

## Results

### TGI modeling

**Figure 1:**
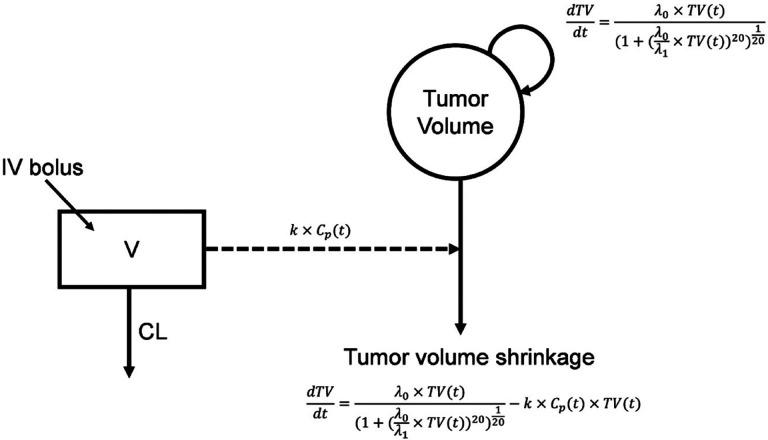
Simeoni TGI model structure.

The Simeoni natural tumor growth model yielded the lowest AIC value (**Table S2**), and was therefore selected as the base model. A linear drug effect was incorporated to describe the drug-induced tumor kill because a nonlinear drug effect led to unstable parameter estimates (**Table S3**). The structure of the Simeoni TGI model is shown in Fig. 1 and in Eq. 1.

[Equation 1] $$\frac{\text{dTV}}{\text{dt}}=\frac{\lambda_{0}\times \text{TV}(t)}{{\left(1+{\left(\frac{\lambda_{0}}{\lambda_{1}}\times \text{TV}(t)\right)}^{20}\right)}^{\frac{1}{20}}}-k\times C_{p}(t)\times \text{TV}(t)$$

where t is time (hours), TV is tumor volume (mm^3^), ${\text{TV}}_{0}$ is TV at t=0, $\lambda_{0}$ and $\lambda_{1}$ represent the exponential and linear tumor growth rate parameters, k is the drug-induced tumor cell kill rate constant, and $C_{p}$ is the simulated plasma concentration based on the PK model. The ${\text{TV}}_{0}$ was included in the model as a regressor.

The final estimates of the population parameters are summarized in Table 1. All TGI parameters were precisely estimated, as indicated by low relative standard errors (RSE < 15%).

**Figure 2:**
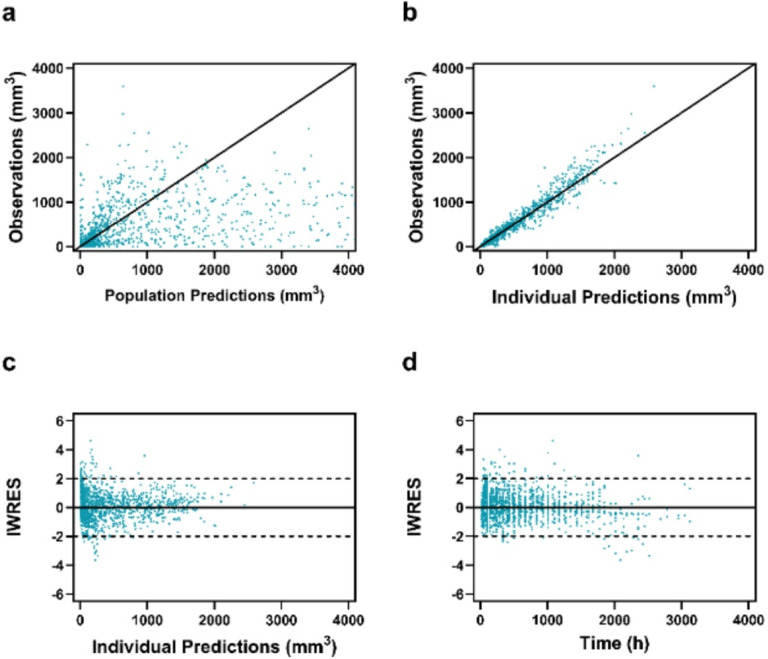
Goodness-of-fit plots of TGI model. Panel a and b show the tumor volume observations versus population and individual predictions, respectively. Panel c and d show individual weighted residuals versus individual predictions and time, respectively.

GOF plots indicated adequate description of the data (Fig. 2). The population predictions showed a wider scatter around the line of unity due to high IIV for the natural growth parameters (Fig. 2a). However, the individual predictions showed strong agreement with the observed tumor volumes (Fig. 2b). Individual weighted residuals (IWRES) were randomly distributed around the line originating at y=0 with no apparent bias over time or individual predicted tumor volumes (Fig. 2c & 2d).

**Figure 3:**
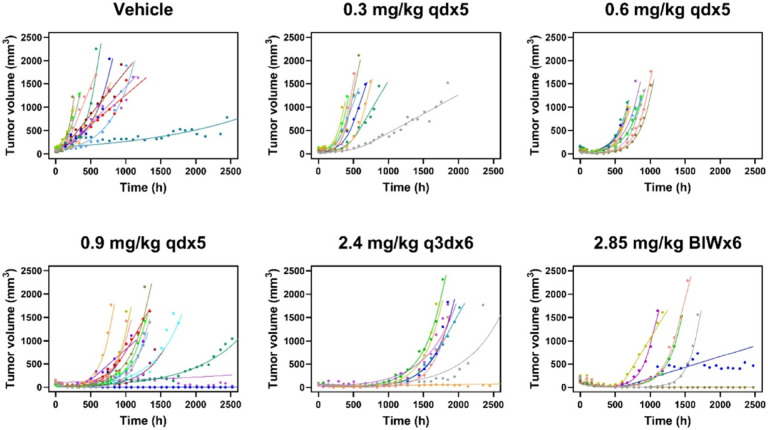
Individual tumor volume fits using post hoc TGI estimates across treatment groups. Solid lines represent individual fits and symbols represent observed tumor volumes.

Furthermore, the individual model fits demonstrated that the model successfully captured the variability in tumor growth profiles of individual mice across treatment groups (Fig. 3). Overall, the individual post-hoc estimates of TGI parameters were robust and precisely estimated with acceptable shrinkage (Table 1).

### TTE modeling

The log-logistic TTE model best described the survival dataset, as it had the lowest AIC value (**Table S4**) and was therefore selected as the base model. The hazard function of the log-logistic TTE model is shown in Eq. 2.

[Equation 2] $$h(t)=\frac{\frac{s}{T_{e}}\times {\left(\frac{t}{T_{e}}\right)}^{\left(s-1\right)}}{1+{\left(\frac{t}{T_{e}}\right)}^{s}}$$

where, h is hazard, t is time (hours), $T_{e}$ is scale parameter representing median survival time, and s is shape parameter (fixed to 4). As shown in Eq. 3, $\lambda_{0},\lambda_{1}$ and $C_{\text{avg}}$ were identified as significant covariates on $T_{e}$. Where $T_{e_{\text{pop}}}$ is the typical population-scale parameter, β represents the coefficient for the respective covariate, normalized by their respective median values, and η is the random effect representing the residual inter-individual variability in the scale parameter.


[Equation 3] $$T_{e_{i}}=T_{e_{\text{pop}}}\times \text{exp}\left[\left(\beta_{\lambda_{0}}\times (\frac{\lambda_{0_{i}}}{\text{median}\;\lambda_{0}}-1)\right)\right]\times \text{exp}\left[\left(\beta_{\lambda_{1}}\times (\frac{\lambda_{1_{i}}}{\text{median}\;\lambda_{1}}-1)\right)\right]\times \text{exp}\left[\left(\beta_{C_{\text{avg}}}\times \frac{C_{\text{avg}}}{\text{median}\;C_{\text{avg}}}\right)\right]\times \text{exp}\left(\eta_{i}\right)$$

The final TTE model parameter estimates are summarized in Table 2. All parameters were estimated with high precision, as indicated by low relative standard errors (RSE < 11%). The final model demonstrated that higher tumor growth rates ($\lambda_{0}$ and $\lambda_{1}$) were associated with shorter survival (β=−0.75 and −0.46, respectively), whereas higher drug exposure ($C_{\text{avg}}$) was associated with longer survival (β=0.63).

**Figure 4:**
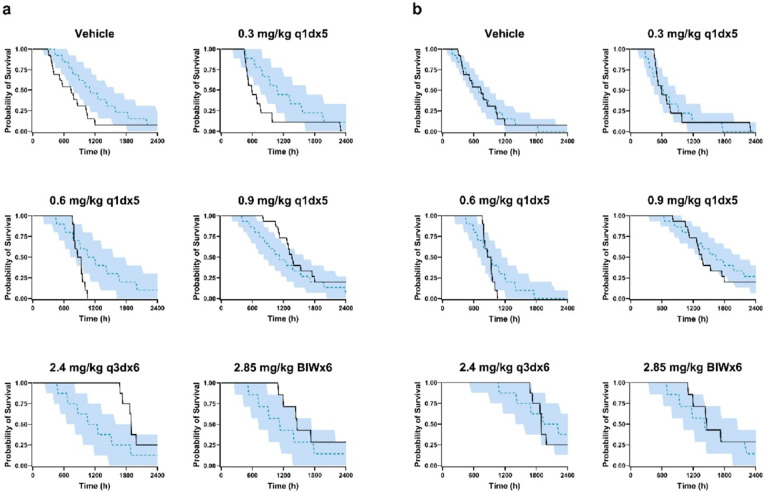
VPC plots of TTE model. Panel a and b show dose-stratified VPC plots of base and final TTE models, respectively. Black solid lines represent observed Kaplan-Meier curves. Blue dashed lines and shaded areas represent model predicted median Kaplan-Meier curves and 90% prediction interval.

VPCs of the base TTE model without covariates captured the overall Kaplan-Meier survival curve; however, they failed to describe the Kaplan-Meier survival curves for individual treatment groups (Fig. 4a). In contrast, the addition of covariates in the scale parameter of the TTE model successfully captured the observed Kaplan-Meier survival curves across all treatment groups (Fig. 4b, Table 2). Moreover, observed survival probabilities were within the 90% prediction intervals derived from the model simulations.

## Discussion

A quantitative understanding of the relationship between tumor growth dynamics and survival is critical for optimizing dosing strategies for anti-cancer agents [18]. Although TGI–TTE modeling frameworks have been extensively applied in clinical settings to describe the relationship between longitudinal tumor size and survival outcomes [19–22], their application to preclinical outcomes remains limited. In this study, using tumor volume and survival data collected from *in vivo* tumor growth inhibition experiments conducted in TC-32 tumor-bearing female athymic nude mice, we developed a translational TGI-TTE modeling framework to quantitatively link drug exposure, tumor growth dynamics, and survival endpoints for MTMSA-Trp.

To characterize natural tumor growth dynamics, several models were explored, and a Simeoni natural tumor growth model was selected as the base model because it provided the best fit, as indicated by the lowest AIC, consistent with previous reports for a similar tumor type [2]. A linear drug effect was subsequently incorporated to describe the MTMSA-Trp-induced tumor cell kill. Incorporation of a linear drug-effect term into the final TGI model implied that MTMSA-Trp exerts a concentration-dependent tumor kill proportional to its plasma concentration. Non-linear drug effect (i.e., Michaelis-Menten) was also explored. However, it led to unstable parameter estimates and was therefore not retained. The linear drug effect successfully captured variability in tumor growth profiles across treatment groups in individual mice. The individual post-hoc estimates of the TGI parameters were robust and precisely estimated with acceptable shrinkage, suggesting the model was suitable for its intended purpose of estimating individual TGI parameters to support subsequent TTE modeling.

For the survival analysis, a parametric log-logistic TTE model best described the survival dataset and was selected as the base model. However, the base TTE model failed to capture the distinct Kaplan-Meier curves across treatment groups, as shown in the dose-stratified diagnostic plots. Building on evidence from clinical TGI–TTE analyses, covariate modeling was conducted to test TGI and PK parameters as covariates in the TTE model. In covariate modeling, tumor growth rates and regimen-specific average concentration ($C_{\text{avg}}$) were identified as significant covariates on the scale parameter of the TTE model. The addition of covariates significantly improved the TTE model, as evidenced by a large drop in the objective function value (Δ-2LL=−87.75). The final TTE model demonstrated that higher tumor growth rates were significantly associated with shorter survival. Conversely, an increased $C_{\text{avg}}$ was significantly associated with longer survival. Parameter estimates in the final TTE model were precise, with relative standard errors below 20%. Because $C_{\text{avg}}$ was defined at the regimen level, the observed exposure-survival relationship should be interpreted as a between-regimen association rather than an individual-level exposure-response relationship.

Model evaluation illustrates the effect of adding covariates on the final TTE model fit. While the base TTE model failed to capture the dose-stratified observed Kaplan-Meier curves, incorporating the covariates into the scale parameter substantially improved the fit by capturing the observed Kaplan-Meier survival curves across all treatment groups. Moreover, observed survival probabilities fell within the 90% prediction intervals derived from the model simulations. Finally, bootstrap resampling confirmed model stability, verifying the robustness of all parameter estimates.

In conclusion, this work establishes a translational TGI–TTE modeling framework that quantitatively links exposure, tumor dynamics, and survival in mouse xenograft studies. This framework complements conventional survival analyses by integrating tumor growth dynamics and drug exposure into a parametric survival modeling framework and supports future study design by enabling simulation-based evaluation of dose regimens in preclinical oncology drug development.

## Supplementary Material

Supplementary Files

This is a list of supplementary files associated with this preprint. Click to download.

- SupplementaryMaterialsNiloyetal.docx

## Figures and Tables

**Table 1:** TGI model parameters

Parameter	Definition	Estimate (%RSE)	IIV (%CV) [shrinkage]	Bootstrap-derived median (95% CI)
$\lambda_{0}$	Exponential growth rate (1/h)	0.0063 (8.56)	0.85 (102.82) [6.12%]	0.0066 (0.0056–0.0076)
$\lambda_{1}$	Linear growth rate (mm^3^/h)	5.93 (11.8)	0.73 (83.39) [28.8%]	5.88 (4.85–8.36)
k	Tumor kill rate (ml/ng/h)	0.000024 (13.4)	0.87 (106.68) [30%]	0.000024 (0.000018–0.000032)
a	Additive residual error (mm^3^/h)	11.22	-	-
b	Proportional residual error (%)	0.22	-	-

**Table:** 

Parameter	Definition	Estimate (%RSE)	IIV (%CV)	Bootstrap-derived median (95% CI)
$T_{e}$	Scale	703.17 (4.72)	0.043 (4.26)	724.59 (602.79–890.34)
$\beta_{\lambda_{0}}$	Coefficient of $\lambda_{0}$	−0.75 (8.3)	-	−0.75 (−1.02 – −0.49)
$\beta_{\lambda_{1}}$	Coefficient of $\lambda_{1}$	−0.46 (17.9)	-	−0.39 (−0.71 – −0.04)
$\beta_{C_{\text{avg}}}$	Coefficient of ${\text{C}}_{\text{avg}}$	0.63 (10.4)	-	0.62 (0.37–0.85)
s	Shape	4 (fixed)	-	-

## Data Availability

Inquiries regarding the datasets should be directed to the corresponding author.
